# Assessment of Venous Thromboembolism Risk Among Patients Admitted to Intensive Care Units in Dhaka City: A Cross‐Sectional Study

**DOI:** 10.1002/puh2.70257

**Published:** 2026-04-27

**Authors:** Md. Shek Sady Khan, Md. Maruf Parves, Abdullah Al Noman, Pradip Kumar Sen Gupta, Md. Mojibur Rahman, Kazi Fharia Tanjim, Masuma Khatun, Muhammad Jubaer Chowdhury, Kazi Ashiqur Rahman, A K M Ferdous Rahman, Md. Sadekul Islam

**Affiliations:** ^1^ Department of Epidemiology Bangladesh University of Health Sciences (BUHS) Dhaka Bangladesh; ^2^ School of Pharmacy BRAC University Dhaka Bangladesh; ^3^ Labaid Specialized Hospital Dhaka Bangladesh; ^4^ Directorate General of Health Services (DGHS) Dhaka Bangladesh; ^5^ Department Of Critical Care Medicine Dhaka Medical College Hospital Dhaka Bangladesh; ^6^ Pharmacy Discipline Khulna University Khulna Bangladesh

**Keywords:** critically ill patients, intensive care unit, risk assessment, venous thromboembolism, VTE prophylaxis

## Abstract

**Background:**

Despite recognized evidence that venous thromboembolism (VTE) is a common and preventable complication in critically ill patients with pulmonary embolism as the most preventable cause of in‐hospital death, we assessed VTE risk and prophylaxis use in ICU patients from Dhaka City.

**Methods:**

This cross‐sectional study was conducted from January to June 2018 in two tertiary care hospitals of Dhaka. We prospectively included 130 patients with ICU admission for ≥24 h. The Padua risk assessment model (RAM) was used to assess VTE risk and patients with a score of ≥4 were classified as high risk. Chi‐square tests and Student's *t*‐tests were used for statistical analysis.

**Results:**

High‐risk status was significantly associated with advancing age and specific clinical predictors. Strong predictors for high‐risk status included thrombophilia, immobilization and active malignancy. Despite high‐risk status, only 28.57% received prophylaxis. Clinicians preferentially administered prophylaxis to elderly patients and those with acute infections, while overlooking predictors like malignancy. The overall in‐hospital mortality was 15.4%.

**Conclusion:**

Nearly half of ICU patients are at high risk for VTE, yet prophylaxis utilization remains inadequate. Systematic implementation of mandatory risk assessment protocols is essential to bridge this clinical gap.

**Clinical Trial Registration:**

This study is a cross‐sectional observational study involving no experimental intervention, treatment allocation or clinical trial procedures. Accordingly, clinical trial registration was not required or applicable for this study design.

## Graphical abstract

Nearly half of ICU patients in Dhaka City are at high risk for venous thromboembolism by Padua risk assessment model, yet only 28.57% of high‐risk patients received prophylaxis. Mandatory systematic risk assessment and LMWH protocols are urgently needed to close this preventable care gap.

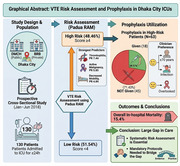

## 1 Introduction

Venous thromboembolism (VTE) incorporating deep vein thrombosis (DVT) and pulmonary embolism (PE) is recognized as a significant healthcare burden worldwide. In the United Kingdom, hospital‐acquired VTE is estimated to cause 25,000–32,000 deaths annually (data based on 2005 England benchmarks), a figure exceeding the combined mortality of breast cancer, AIDS and road traffic accidents [1, 2]. There is no currently universally validated standardized VTE risk assessment model (RAM) designed for critically ill patients, although these population has a significantly higher VTE risk because of several contributing factors.

In Bangladesh, the true disease burden is often under‐recognized due to limited systematic screening and low clinical awareness. Recent local evidence suggests a substantial prevalence that challenges earlier assumptions of low risk in Asian populations. For instance, a prospective study conducted at Dhaka Medical College Hospital between 2022 and 2023 identified that 32.07% of acute stroke patients presented with DVT [3]. Furthermore, in local surgical settings, the incidence of DVT reaches 12.5% in patients undergoing major abdominal surgeries lasting longer than 2 h [4]. Implementation of prophylaxis in Bangladeshi ICUs is hindered by systemic barriers, including the absence of mandatory institutional protocols and a significant ‘fear of bleeding’ among clinicians, which frequently leads to the omission of indicated pharmacological agents [5].

Critically ill patients have a high risk of VTE because they are susceptible to general risk factors of VTE as well as those specific to ICU patients, such as sedation, immobilization or vasopressors [6]. The incidence varies widely across populations, with documented annual rates per 100,000 individuals of 23 in Caucasians, 29 in African Americans, 14 in Hispanics and only 6 (the lowest rate) among Asian‐Pacific Islanders [7]. Nonetheless, VTE occurrence rates were 3.7% in the ICU versus 1.2% in the non‐ICU group; constituting a three‐fold larger risk among ICU patients with critically ill status [8].

DVT can develop in up to 81% of critically ill patients if no thromboprophylaxis is used, with more than 44% developing DVT after receiving prophylatic doses [9]. In ICU patients with high adherence to chemoprophylaxis, 1.4% had symptomatic VTE that was recognized clinically (3%–7%, different studies) [10, 11]. PE results in 5%–10% of hospital deaths; hence, VTE is the predominant preventable cause of in‐hospital death [12].

Although consensus guidelines based on evidence have existed for close to 2 decades, prophylaxis is grossly underutilized worldwide [13]. Qualitative data from individual studies or countries have documented compliance, but the proportion of at risk patients receiving appropriate prophylaxis on a global scale has not been clearly established [14]. Additionally, this global knowledge gap is more likely to be substantial in low‐ and middle‐income countries (LMICs) where VTE awareness may also be limited among healthcare providers.

This study aims to determine VTE risk among ICU patients using validated assessment tools, describe prevalent predictors and evaluate current prophylactic practices compared to international standards.

## 2 Rationale

VTE management poses several unique challenges in a country like Bangladesh which is resource limited as most LMICs. The paucity of local literature on VTE incidence and risk factors has left clinicians reliant on Western literature for guideline‐directed VTE prophylaxis. Consequently, reliance on Western guidelines may inadequately reflect the true disease burden and healthcare context in Bangladesh [15]. Recent reports from Asian countries indicate the rate of VTE is even higher than previously reported among Asian populations [16].

## 3 Methods

### 3.1 Study Design and Setting

This cross‐sectional analytical study was conducted in the ICUs of two tertiary care centres in Dhaka City, Bangladesh, including (1) Dhaka Medical College Hospital (public sector) and (2) BRB Hospitals Limited (private sector), between January and June 2018. Following the Patanwala guides for medical record reviews, we utilized a standardized abstraction tool to minimize inter‐rater variability and consecutive sampling to ensure a representative cohort [17]. The study protocol was approved by the Ethical Review Committee (ERC) of the Bangladesh University of Health Sciences (BUHS), Dhaka, Bangladesh (Approval Reference: BUHS/BIO/EA/19/205; Date: 06 August 2019).

### 3.2 Participants

**Inclusion criteria**:

- Age ≥18 years
- ICU admission duration ≥24 h
- Complete medical records available
- Informed consent obtained

**Exclusion criteria**:

- Pre‐existing anticoagulant therapy
- ICU admission specifically for VTE treatment
- Incomplete essential data for risk assessment
- Declined participation or inability to provide consent

### 3.3 Sample Size Calculation

Sample size was calculated using the formula:

$$n=\frac{z^{2}pq}{d^{2}}$$

where *z* = 1.96 (95% CI), *p* = 0.25 (assumed VTE prevalence), *q* = 0.75 and *d* = 0.05. Calculated sample size: *N* = 148. Accounting for 10% attrition: final sample size = 167. Actual recruitment: 130 patients (after exclusions).

### 3.4 Data Collection

Structured interviews, clinical assessments and review of medical records were used to gather data at the point of admission. We assessed VTE risk factors using an 11‐item questionnaire with established validity. Patients were risk stratified for VTE by Padua Prediction Score with scores ≥4 considered high‐risk. Predictors were defined following the original Barbar criteria [18]: reduced mobility (bed rest with bathroom privileges for ≥3 days), active cancer (metastases or treatment within the last 6 months) and thrombophilia (documented genetic or acquired clotting defects). Prophylaxis utilization was defined as the administration of pharmacological agents (e.g., LMWH) or mechanical devices (e.g., IPC).

### 3.5 Statistical Analysis

Descriptive statistics, including means, SDs and percentages, were computed. Categorical variables were analysed using chi‐square tests (with Yates correction), whereas continuous ones with Student´s *t*‐test. Given the cross‐sectional design and high prevalence of outcomes (>10%), prevalence ratios (PR) were calculated to avoid the overestimation of associations inherent in odds ratios (OR). *p* Values less than 0.05 were considered statistically significant. Descriptive analysis was conducted by SPSS version 25.0, whereas PR modelling and confidence interval estimations were performed using Stata version 14.2.

### 3.6 Ethical Considerations

This study was approved by the Ethical Review Committee (ERC) of the Bangladesh University of Health Sciences (BUHS), Dhaka, Bangladesh (Approval Reference No.: BUHS/BIO/EA/19/205; Date: 06 August 2019). Verbal informed consent was obtained from all competent subjects prior to enrolment, as approved by the ERC for this study design. For patients who were incapacitated (e.g., sedated or mechanically ventilated), verbal consent was obtained from the next of kin or legal guardian in accordance with the ERC guidelines. Patients remained anonymous throughout this study; all data were coded and identifying information was not recorded in the analysis dataset. Participation was entirely voluntary and there were no penalties for withdrawal at any time. The study was conducted in accordance with the Declaration of Helsinki and the ethical guidelines issued by the BUHS ERC.

## 4 Results

### 4.1 Patient Characteristics

**FIGURE 1:**
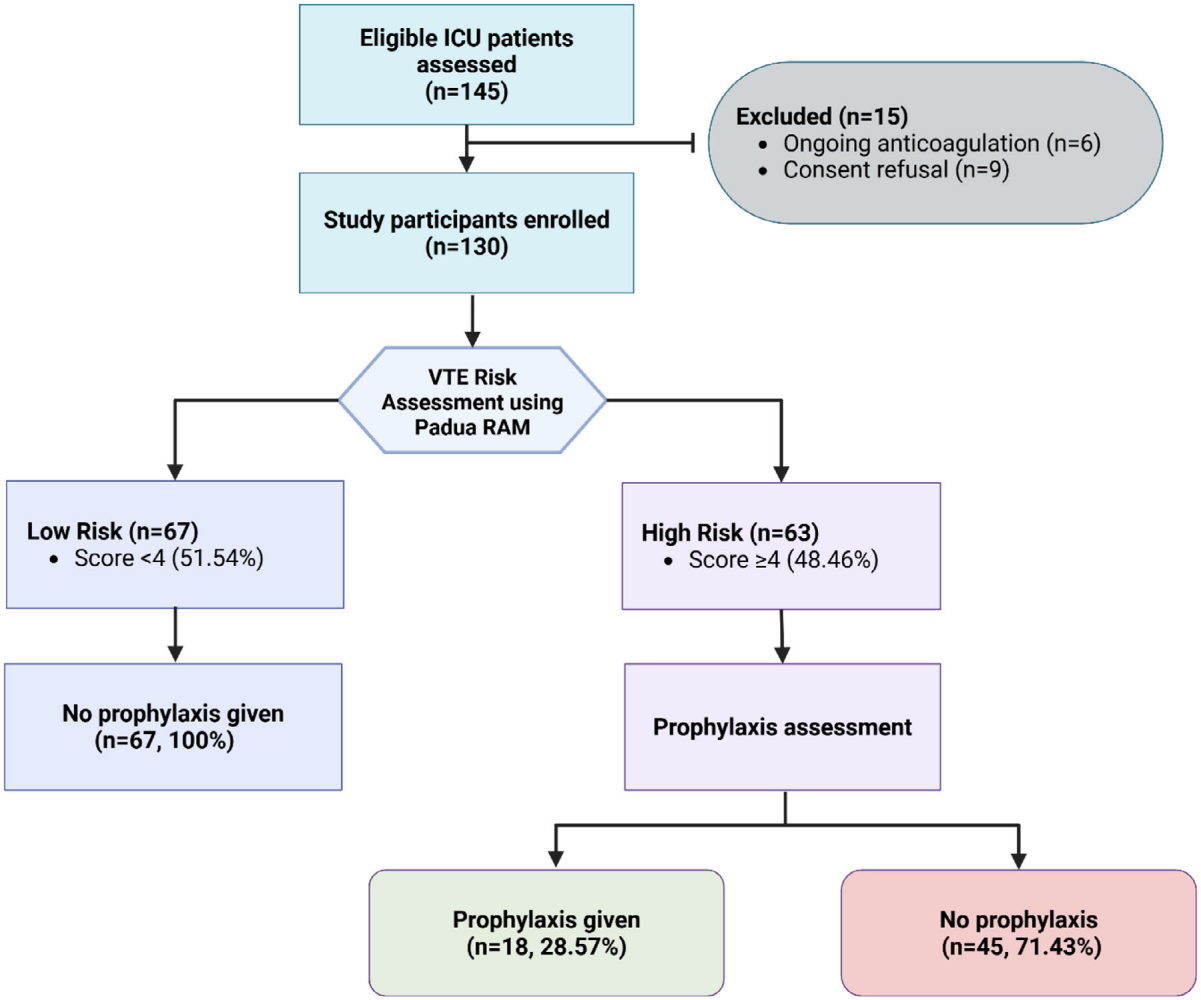
Patient flow diagram. RAM, risk assessment model; VTE, venous thromboembolism.

Of 145 eligible patients, 15 were excluded (6 because they remained on anticoagulants and 9 declined to participate), with a final cohort of 130 study participants (Figure 1).

Table 1 shows the baseline characteristics. In the present study, the mean age was 48.38 ± 20.93 years with 75 (57.69%) males and 55 (42.31%) females.

**TABLE 1:** Baseline characteristics of study participants (*N* = 130).

Characteristic	All patients (*N* = 130)	Low risk (*N* = 67)	High risk (*N* = 63)	*p* value
Age, years (mean ± SD)	48.38 ± 20.93	41.21 ± 19.44	56.02 ± 19.87	<0.01^*^
Male sex, *n* (%)	75 (57.69)	36 (53.73)	39 (61.90)	0.35
Female sex, *n* (%)	55 (42.31)	31 (46.27)	24 (38.10)	0.35
ICU stay, days (mean ± SD)	5.2 ± 3.8	4.8 ± 3.2	5.7 ± 4.3	0.21
Hospital setting				
‐ Public (DMCH), *n* (%)	78 (60.0)	41 (61.2)	37 (58.7)	0.77
‐ Private (BRB), *n* (%)	52 (40.0)	26 (38.8)	26 (41.3)	0.77

*Note:* BRB: BRB Hospitals Limited.

Abbreviation: DMCH, Dhaka Medical College Hospital.

* Statistically significant (*p* < 0.05).

### 4.2 VTE Risk Assessment

**FIGURE 2:**
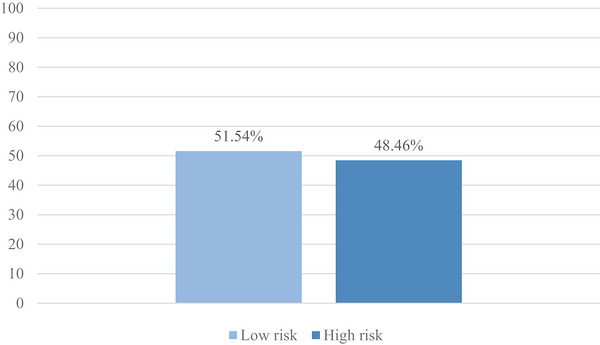
Distribution of VTE Risk categories.

Using the Padua RAM (Table 2), 63 patients (48.46%) were high‐risk (score ≥4) and 67 were low‐risk (score <4) (Figure 2).

**TABLE 2:** Padua risk assessment model for VTE risk stratification.

Risk factor	Score
Prior episode of VTE	3
Thrombophilia	3
Decreased mobility	3
Active malignancy	3
Previous trauma or surgery (within 1 month)	2
Age ≥70 years	1
Heart and/or respiratory failure	1
Ischaemic stroke or acute myocardial infarction	1
Acute rheumatologic disorder and/or acute infection	1
Obesity (BMI >30 kg/m^2^)	1
Hormonal therapy	1

*Note:* **Risk classification**: low risk: <4 points; high risk: ≥4 Points.

Abbreviations: BMI, body mass index; VTE, venous thromboembolism.

Age of the high‐risk group was significantly higher than that of the low‐risk group (56.02 ± 19.87 vs. 41.21 ± 19.44, *p* < 0.01). In patients at high risk, 39 (61.90%) were male and 24 (38.10%) female.

### 4.3 Risk Factor Analysis

Table 3 presents the comprehensive analysis of predictors associated with high VTE risk. Several factors showed significant associations with high VTE risk including thrombophilia (22.22% vs. 2.99%; PR 7.44, 95% CI 1.76–31.46, *p* < 0.01), decreased mobility (65.08% vs. 13.43%; PR 4.84, 95% CI 2.57–9.14, *p* < 0.01), active malignancy (34.92% vs. 5.97%; PR 5.85, 95% CI 2.13–16.03, *p* < 0.01), ischaemic stroke/acute MI (38.10% vs. 17.91%; PR 2.13, 95% CI 1.17–3.88, *p* = 0.01) and obesity with BMI >30 (26.98% vs. 8.96%; PR 3.01, 95% CI 1.27–7.16, *p* < 0.01). The strongest associations based on magnitude were observed with thrombophilia, followed by active malignancy and decreased mobility.

**TABLE 3:** Risk factors associated with high VTE risk (*N* = 130).

Variable	Low risk (*N* = 67) *n* (%)	High risk (*N* = 63) *n* (%)	*p* value	PR (95% CI)
Male sex	36 (53.73)	39 (61.90)	0.35	1.15 (0.86–1.55)
Elderly age (≥70 years)	6 (8.96)	13 (20.63)	0.06	2.30 (0.93–5.69)
Prior episode of VTE	1 (1.49)	4 (6.35)	0.15	4.25 (0.49–37.04)
Thrombophilia	2 (2.99)	14 (22.22)	<0.01^*^	7.44 (1.76–31.46)
Decreased mobility	9 (13.43)	41 (65.08)	<0.01^*^	4.84 (2.57–9.14)
Active malignancy	4 (5.97)	22 (34.92)	<0.01^*^	5.85 (2.13–16.03)
Previous trauma/Surgery	9 (13.43)	14 (22.22)	0.19	1.65 (0.77–3.55)
Heart/Respiratory failure	22 (32.84)	25 (39.68)	0.42	1.21 (0.76–1.91)
Ischaemic stroke/Acute MI	12 (17.91)	24 (38.10)	0.01^*^	2.13 (1.17–3.88)
Acute rheumatologic disorder/Infection	19 (28.36)	19 (30.16)	0.82	1.06 (0.62–1.82)
Obesity (BMI >30)	6 (8.96)	17 (26.98)	<0.01^*^	3.01 (1.27–7.16)
Hormonal therapy	2 (2.99)	6 (9.52)	0.12	3.19 (0.67–15.23)

Abbreviations: BMI, body mass index; CI, confidence interval; MI, myocardial infarction; PR, prevalence ratio; VTE, venous thromboembolism.

* Statistically significant (*p* < 0.05).

### 4.4 Prophylaxis Utilization

**FIGURE 3:**
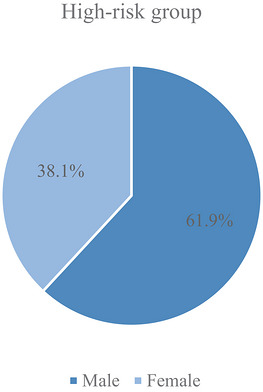
Prophylaxis utilization among high‐risk patients.

Only 18 (28.57%) of the 63 high‐risk patients received VTE prophylaxis, whereas no VTE prophylaxis was administered in 45 cases (71.43%) (Figure 3).

No low‐risk patients received prophylaxis. The prophylaxis status was significantly associated with VTE risk (*p *< 0.01).

### 4.5 Factors Associated With Prophylaxis Use

Table 4 demonstrates factors associated with prophylaxis utilization among high‐risk patients. Factors significantly associated with prophylaxis use included elderly age <70 years (38.89% vs. 13.33%; PR 4.33, 95% CI 1.25–15.0, *p* = 0.02), decreased mobility (88.89% vs. 55.56%; PR 6.40, 95% CI 1.37–29.9, *p* = 0.01) and acute rheumatologic disorder/infection (61.11% vs. 17.78%; PR 7.33, 95% CI 2.28–23.6, *p* < 0.01). High‐risk patients receiving prophylaxis had higher mean RAM scores than those not receiving prophylaxis (6.61 ± 1.91 vs. 5.67 ± 2.06, *p* = 0.09), though this difference was not statistically significant.

**TABLE 4:** Factors associated with prophylaxis use among high‐risk patients (*N* = 63).

Variable	Prophylaxis given (*N* = 18) *n* (%)	No prophylaxis (*N* = 45) *n* (%)	*p* value	PR (95% CI)
Male sex	12 (66.67)	27 (60.00)	0.62	2.39 (1.33–4.30)
Elderly age (≥70 years)	7 (38.89)	6 (13.33)	0.02^*^	0.91 (0.32–2.57)
Prior episode of VTE	0 (0)	4 (8.89)	0.19	—
Thrombophilia	3 (16.67)	11 (24.44)	0.50	3.90 (1.14–13.33)
Decreased mobility	16 (88.89)	25 (55.56)	0.01^*^	1.66 (0.98–2.81)
Active malignancy	6 (33.33)	16 (35.56)	0.87	2.84 (1.18–6.79)
Previous trauma/Surgery	4 (22.22)	10 (22.22)	1.00	2.66 (0.88–8.05)
Heart/Respiratory failure	9 (50.00)	16 (35.56)	0.29	1.89 (0.90–3.97)
Ischaemic stroke/Acute MI	8 (44.44)	16 (35.56)	0.51	2.13 (0.98–4.62)
Acute rheumatologic disorder/Infection	11 (61.11)	8 (17.78)	<0.01^*^	0.77 (0.33–1.80)
Obesity (BMI >30)	2 (11.11)	15 (33.33)	0.07	7.98 (1.90–33.49)
Hormonal therapy	0 (0)	6 (13.33)	0.10	—
Mean RAM score ± SD	6.61 ± 1.91	5.67 ± 2.06	0.09	—

Abbreviations: BMI, body mass index; CI, confidence interval; MI, myocardial infarction; PR, prevalence ratio; RAM, risk assessment model; VTE, venous thromboembolism.

* Statistically significant (*p *< 0.05).

## 5 Discussion

### 5.1 Principal Findings

The current study suggests that approximately 48.46% patients in ICU of Dhaka City are at high risk of VTE which is compatible with international data from ENDORSE, representing a largely similar proportion of hospitalized patient population at VTE risk [19]. Nonetheless, just 28.57% of our high‐risk patients received prophylaxis, which is somewhat more concerning and at the very low end of previously reported usage rates (13%–64%) [20].

### 5.2 Comparison With International Data

Recent international studies demonstrate substantial variability in VTE incidence among ICU patients, with reported rates ranging from clinically diagnosed symptomatic VTE of 1.4% in patients with high adherence to chemoprophylaxis to higher overall VTE incidence of 7% in specific ICU populations [21, 22]. A comprehensive meta‐analysis of 42 studies comprising 27,344 ICU patients revealed an overall pooled prevalence of VTE of 10.0% (95% CI: 7.0%–14.0%), with rates varying significantly based on thromboprophylaxis strategy, patient severity and screening protocols [23]. Our findings align with global trends showing substantial underutilization of appropriate thromboprophylaxis despite available evidence‐based guidelines. The landmark ENDORSE study demonstrated marked international variations in prophylaxis utilization, with only 50.2% of at‐risk patients worldwide receiving appropriate VTE prophylaxis, attributed to factors including physician awareness, guideline availability, educational factors, reimbursement structures and healthcare resource allocation [24]. These factors are particularly relevant in resource‐limited settings like Bangladesh.

### 5.3 Clinical Implications

The findings suggest several critical VTE prevention shortfalls that require urgent address. Validated risk assessment tools exist; however, the systematic approach to VTE risk evaluation appears not to be well established within healthcare systems and may lead patient susceptible to preventable complications. Of those 66 patients who met high risk criteria, only about one in four received prophylaxis (28.57%), a shockingly low rate that represents an enormous gap in VTE morbidity and mortality prevention for a population considered at high risk of VTE and suggests a profound failure to translate evidence‐based recommendations into clinical practice. Notwithstanding, the selective prophylaxis patterns identified were mainly concentrated in patients with reduced mobility and acute infections, indicating that clinicians appear to address a few surviving recognized risk factors whilst omitting others meriting equivalent notice.

### 5.4 Recent Guideline Updates

As recently as 2024 the American College of Chest Physicians (CHEST) has updated their antithrombotic therapy guidelines, highlighting the ongoing VTE management algorithms evolution [25]. Current guidelines emphasize that hospitalization for acute medical illness reflects an important point of contact in which to apply VTE prophylaxis, rendering our findings timely and pertinent to practice recommendations [26].

### 5.5 Local Context and Barriers

A combination of factors may have contributed to poor utilization of prophylaxis in the health‐system‐level practice and these factors are interconnected. This results in substantial underrecognition of the VTE risk by healthcare providers and an inability to provide prophylaxis in a resource‐constrained area, further complicating the delivery of care. Not only does the lack of mandatory institutional protocols not help, but the perceived threat of bleeding complications and educational gaps related to VTE risk assessment are further influencing patterns in physician practice.

### 5.6 Implications for Clinical Practice

Portable compression ultrasonography for DVT screening is technically feasible at both study hospitals but is not currently deployed as a routine ICU surveillance tool, primarily due to cost constraints and limited availability of trained vascular sonographers. Implementing systematic bedside ultrasonography at ICU admission would allow earlier identification of subclinical DVT events and represents an achievable quality improvement target that does not require major capital investment. On the basis of published meta‐analytic evidence, systematic pharmacological thromboprophylaxis in medical ICU patients is associated with approximately a 40%–50% relative risk reduction in symptomatic VTE events. Quantifying the absolute mortality reduction attributable to prophylaxis in our specific cohort is beyond the scope of this cross‐sectional study; however, given the 15.4% in‐hospital mortality observed and the high‐risk proportion (48.46%), even a modest reduction in VTE‐related deaths would represent substantial clinical benefit. With regard to bleeding risk, current meta‐analyses estimate that LMWH prophylaxis is associated with an absolute increase in major bleeding of approximately 0.4%–0.8% in medical ICU patients a risk that does not offset the substantial thrombotic benefit in appropriately identified high‐risk patients, per current CHEST guideline recommendations.

### 5.7 Strengths and Limitations

To our knowledge, this study represents the first systematic implementation and validation of the Padua RAM for VTE risk assessment in a Bangladeshi ICU setting. The inclusion of public and private sector counterparts confers generalizability to our findings; meanwhile, a thorough risk assessment provided insight on common patterns of the disease in local settings. Several limitations, however, should be acknowledged: The cross‐sectional design makes it impossible to determine temporal associations; this was a multi‐centre study conducted at two tertiary hospitals and the generalizability of the findings could be limited by geographic restriction to Dhaka City; there may not have been sufficient power in our relatively small sample size; and a routine VTE diagnostic work‐up (e.g., compression ultrasonography) was not systematically performed, meaning that asymptomatic VTE events may have been under‐ascertained. Moreover, the usage of medical record data collection may produce an information bias and we cannot evaluate long‐term outcomes that could affect clinical impact.

## 6 Conclusions and Future Directions

This study reveals a significant and concerning gap in the care of critically ill patients in Dhaka. Nearly half (48.46%) of the ICU population was identified as being at high clinical risk for VTE, yet only 28.57% received appropriate prophylaxis. The strongest predictors of high‐risk status, by PR, were thrombophilia (PR 7.44), active malignancy (PR 5.85) and decreased mobility (PR 4.84) yet clinicians disproportionately directed prophylaxis towards patients with reduced mobility and acute infections, while overlooking malignancy and thrombophilia.

The evidence is not yet being translated into bedside practice. Systemic barriers the absence of mandatory hospital protocols and a prevailing fear of bleeding among clinicians must be addressed through institutional adoption of the Padua RAM as a standard admission tool, integration of clinical decision support and a cultural shift in medical education that positions VTE prevention as a core patient safety priority.

Future research should include prospective multi‐centre studies with systematic VTE screening and severity scoring (APACHE/SOFA) to quantify the absolute mortality reduction achievable with protocol‐driven prophylaxis in Bangladeshi ICUs.

## Author Contributions

**Ferdous Rahman**: data curation, formal analysis. **Abdullah Al Noman**: investigation, validation, formal analysis, supervision, methodology, project administration, writing – review and editing, writing – original draft. **Md. Maruf Parves**: software, writing – review and editing, visualization, supervision, methodology, resources, project administration, writing – original draft. **Pradip Kumar Sen Gupta**: visualization, data curation, resources. **Md. Shek Sady Khan**: conceptualization, methodology, writing – original draft, funding acquisition. **Md. Mojibur Rahman**: writing – review and editing, resources, investigation. **Kazi Ashiqur Rahman**: data curation, supervision, resources. **Md. Sadekul Islam**: software, data curation, resources. **Kazi Fharia Tanjim**: investigation, data curation, resources. **Muhammad Jubaer Chowdhury**: validation, supervision, resources. **Masuma Khatun**: resources, writing – review and editing.

## Funding

This study was conducted as part of the Master of Public Health program at Bangladesh University of Health Sciences. No external funding was received.

## Conflicts of Interest

The authors declare no conflicts of interest.

## Data Availability

The data that support the findings of this study are available from the corresponding author upon reasonable request.
